# Association Between Immune-Related Adverse Events During Anti–PD-1 Therapy and Tumor Mutational Burden

**DOI:** 10.1001/jamaoncol.2019.3221

**Published:** 2019-08-22

**Authors:** David Bomze, Omar Hasan Ali, Andrew Bate, Lukas Flatz

**Affiliations:** 1 Institute of Immunobiology, Kantonsspital St Gallen, St Gallen, Switzerland; 2 Department of Dermatology, University Hospital Zurich, Zurich, Switzerland; 3 Division of Translational Medicine, Department of Medicine, NYU School of Medicine, New York, New York

## Abstract

This database study compares surveillance data on immune-related adverse events (irAEs) in patients treated with immune checkpoint inhibitors to determine whether irAEs experienced during anti–PD-1 therapy correlate with tumor mutational burden across 18 cancer types.

Immune checkpoint inhibitors (ICIs) that target the programmed death 1 receptor (anti–programmed cell death 1 [PD-1] therapy) have ushered in a new era of cancer therapy. However, their application has been curtailed by serious immune-related adverse events (irAEs), such as colitis, pneumonitis, and myocarditis, that remain largely unpredictable. Although the use of tumor mutational burden (TMB) as a biomarker for expected therapy response has been advocated,^1^ a similar parameter for irAEs is lacking. In an attempt to fill this clinically relevant knowledge gap, we investigated the association between irAEs reported during anti–PD-1 therapy and TMB by comparing large-scale surveillance data of irAEs with the median TMB across multiple cancer types.

## Methods

We retrieved postmarketing data of adverse events from the US Food and Drug Administration Adverse Event Reporting System (FAERS) from July 1, 2014, to March 31, 2019. According to the ethics committee policy of the EKOS (Ethikkommission Ostschweiz, Switzerland), this study was exempt from ethical review because all analyzed data sets are deidentified and publicly available. We considered only reports for which the anti–PD-1 agents nivolumab or pembrolizumab were the suspected cause of adverse events. Anti–PD-1 and anti-cytotoxic T-lymphocyte–associated protein 4 combination treatment was excluded. Closely related indications were aggregated to unified terms; for example, “malignant melanoma” was aggregated to “melanoma.” To limit our analysis to irAEs, we filtered terms to match broadly accepted diagnoses that were outlined in peer-reviewed irAE management guidelines. The median TMB in tumor tissue was obtained from previously published comprehensive genomic profiling.^2,3^ Lastly, we only considered cancers for which there were at least 100 cases of adverse events during anti–PD-1 therapy reported in FAERS. To assess the risk of a patient developing any irAE, we estimated reporting odds ratios (RORs) by comparing the odds of reporting these irAEs rather than others for the anti–PD-1 agents with the odds for all other drugs in the database, which represents standard practice for quantitative analyses of data in FAERS and similar databases.^4^

## Results

Our search strategy identified a total of 47 304 adverse events (AEs) in 16 397 patients reported as treated with anti–PD-1 monotherapy for 19 different cancer types. Of these patients, 3661 had at least 1 irAE (22.3%; 95% CI, 21.7-23.0). The comparator group comprised 16 411 749 AE reports from 5 160 064 patients. Our analysis revealed a significant positive correlation between the ROR of reporting an irAE during anti–PD-1 therapy and the corresponding TMB across multiple cancer types, with a higher ROR of irAE associated with a higher median number of coding somatic mutations per megabase of DNA (Figure; Pearson correlation coefficient *R* = 0.704; *P* < .001). The correlation coefficient suggests that 50% of the differences in the irAE risk across cancer types may be attributed to the TMB.

**Figure.**
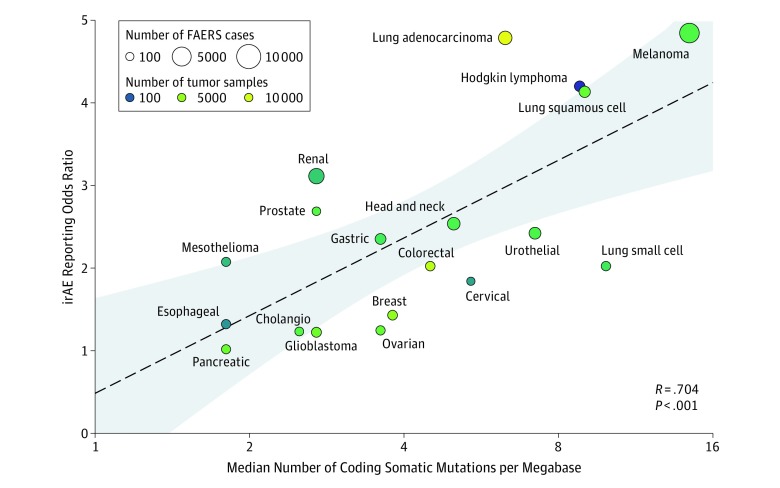
Association of Tumor Mutational Burden With Immune-Related Adverse Events During Anti–PD-1 Therapy Across Multiple Cancers The x-axis indicates the tumor mutational burden (TMB)—defined as the median number of coding somatic mutations per megabase of DNA—across 19 cancer types. Data on the x-axis are presented on a logarithmic scale. The y-axis shows the reporting odds ratio of any immune-related adverse event (irAE) across cancer types, calculated using data from the US Food and Drug Administration Adverse Event Reporting System (FAERS) database. The dashed line represents the 95% CI of the linear fit. Circle size represents the total number of FAERS cases for each cancer type, and the color indicates the total number of tumor samples used to measure TMB for each cancer type.

## Discussion

Our analysis indicates that cancers with a high TMB, such as melanoma and non–small cell lung cancer, are associated with a higher irAE ROR during anti–PD-1 therapy, strongly suggesting that these cancers are associated with a higher risk of irAEs than cancers with a low TMB. A possible explanation for this finding may be the different neoantigenic load across cancer types. Additionally, studies have shown that T cells that react against a neoantigen can crossreact against the corresponding wild-type protein.^5^ Another contributing mechanism may be antigen spreading, where tumor cell death releases antigens, including neoantigens, that prime lymphocytes against the wild-type antigens in healthy tissue. Given the results of the analysis, we propose that the association between irAEs and improved response to anti–PD-1 treatment are linked via an underlying neoantigenic potential that stems from a high TMB. A limitation of the study is the use of spontaneous reports for indirectly measuring the risk of irAE. Furthermore, patients with cancers with a high TMB may receive a longer course of anti–PD-1 treatment. However, most irAEs reported during anti–PD-1 therapy develop within the first few weeks of treatment.^6^ This finding suggests that therapy duration is unlikely to influence the statistical outcome. In conclusion, a high TMB may be a useful biomarker for assessing patients’ risk of irAEs during anti–PD-1 therapy, which has particular relevance for vulnerable patient groups.

## References

[1] Yarchoan M, Hopkins A, Jaffee EM Tumor mutational burden and response rate to PD-1 inhibition. N Engl J Med. 2017;377(25):2500-2501. doi:10.1056/NEJMc1713444 29262275 PMC6549688

[2] Chalmers ZR, Connelly CF, Fabrizio D, Analysis of 100,000 human cancer genomes reveals the landscape of tumor mutational burden. Genome Med. 2017;9(1):34. doi:10.1186/s13073-017-0424-2 28420421 PMC5395719

[3] Liang WS, Vergilio JA, Salhia B, Comprehensive genomic profiling of Hodgkin lymphoma reveals recurrently mutated genes and increased mutation burden. Oncologist. 2019;24(2):219-228. doi:10.1634/theoncologist.2018-0058 30108156 PMC6369943

[4] Bate A, Evans SJ Quantitative signal detection using spontaneous ADR reporting. Pharmacoepidemiol Drug Saf. 2009;18(6):427-436. doi:10.1002/pds.1742 19358225

[5] Castle JC, Kreiter S, Diekmann J, Exploiting the mutanome for tumor vaccination. Cancer Res. 2012;72(5):1081-1091. doi:10.1158/0008-5472.CAN-11-3722 22237626

[6] Martins F, Sofiya L, Sykiotis GP, Adverse effects of immune-checkpoint inhibitors: epidemiology, management and surveillance [published online May 15, 2019]. Nat Rev Clin Oncol. 2019. doi:10.1038/s41571-019-0218-0 31092901

